# Establishment and evaluation of a predictive scoring system for successful decannulation of tracheostomy in neurosurgery patients

**DOI:** 10.3389/fneur.2026.1837392

**Published:** 2026-05-22

**Authors:** Yin Hu, Lifang Mao, Qing Liu, Yanhua Jiang, Shun Li

**Affiliations:** 1 Department of Thoracic Surgery Ward III, Sichuan Cancer Hospital and Institute, Chengdu, Sichuan, China; 2 Department of Nursing, Affiliated Hospital of North Sichuan Medical College, Nanchong, Sichuan, China; 3 Cancer Day-Care Unit, Department of Medical Oncology, Cancer Center, West China Hospital, Sichuan University/West China School of Nursing, Sichuan University, Chengdu, Sichuan, China; 4 Department of Neurosurgery, Affiliated Hospital of North Sichuan Medical College, Nanchong, Sichuan, China

**Keywords:** airway management, decannulation, neurocritical care, neurosurgery, predictive model, prognostic factors, scoring system, tracheostomy

## Abstract

**Objective:**

To establish and evaluate a predictive scoring system for successful decannulation of tracheostomy in neurosurgery patients.

**Methods:**

A prospective observational study was conducted. Patients undergoing tracheostomy in the neurosurgery departments of two tertiary hospitals in Sichuan Province were enrolled and divided into training and validation sets based on admission time. Multivariate logistic regression was used to identify predictive factors for successful decannulation, and a scoring system was developed based on the regression coefficients. Model performance was assessed using area under the receiver operating characteristic curve (AUC), calibration plots, and decision curve analysis. Temporal validation was performed using a later time cohort from the same institutions.

**Results:**

A total of 216 patients were included, with an overall decannulation success rate of 63.9%. Independent predictive factors included supratentorial lesion location (OR 8.05, 95% CI 1.87–34.72), Glasgow Coma Scale (GCS) >8 at decannulation (OR 21.21, 95% CI 4.89–92.04), < 6 airway suctioning episodes/24 h (OR 13.13, 95% CI 3.31–52.09), capping trial ≥24 h (OR 6.28, 95% CI 1.62–24.43), and serum albumin (Alb) ≥35 g/L (OR 4.71, 95% CI 1.43–15.51). The scoring system (0–15 points) achieved an AUC of 0.933 (95% CI: 0.888–0.978) in the training set and 0.930 (95% CI: 0.868–0.993) in the validation set. A cut-off of ≥10 points yielded a sensitivity of 91.9% and specificity of 84.3%.

**Conclusion:**

The predictive scoring system is scientifically valid and clinically practical, providing a simple tool to assist in decannulation decision-making and timing optimization in neurosurgical patients.

## 1 Introduction

Neurosurgical diseases, including cerebrovascular diseases, traumatic brain injury, and intracranial tumors, are characterized by high morbidity, mortality, and disability rates, imposing a heavy disease burden on society and families (1). Patients often require artificial airway support due to impaired consciousness, respiratory failure, and compromised airway protection. When prolonged (>2 weeks) artificial airway support is anticipated, early tracheostomy is recommended (2). The tracheostomy rate in neurosurgical patients has been reported to ranges from 15 to 45% (3–5).

Tracheostomy involves creating an incision in the cervical trachea and inserting a tube to bypass upper airway obstruction, improve respiratory function, and clear lower respiratory tract secretions (6). However, this procedure disrupts airway integrity, resulting in loss of the upper airway mucosa's physiological functions (warming, humidifying, and filtering). It may also lead to complications such as pulmonary infection, tracheal stenosis, and tracheoesophageal fistula (7–9). Therefore, when the patient no longer depends on the tracheostomy tube, early decannulation should be performed to restore spontaneous ventilation. Successful decannulation is an important marker of clinical improvement, yet decannulation failure rates are as high as 40% to 60% (10–14). Long-term cannulation impairs swallowing and verbal communication, causes psychological distress, and increases caregiver burden (15–17).

Clinical predictive models quantify individual disease risk or prognosis using mathematical models (18, 19). Scoring systems, as simplified forms of predictive models, achieve rapid risk assessment by converting regression coefficients into intuitive scores, becoming important decision-support tools in clinical practice.

Based on the clinical need for early decannulation in neurosurgical patients, this study aimed to construct a predictive scoring system for successful tracheostomy decannulation through a prospective observational study, providing a simple and reliable tool to support clinical decision-making.

## 2 Materials and methods

### 2.1 Study subjects

Patients diagnosed with cerebrovascular disease, traumatic brain injury, or brain tumor who underwent tracheostomy in the neurosurgery departments of two tertiary hospitals in Sichuan Province were selected. Based on admission time, they were divided into a training set (February 2023–August 2023) and a validation set (September 2023–November 2023). Inclusion criteria were: age ≥18 years; confirmed diagnosis by imaging or pathology; first-time tracheostomy; informed consent provided. Exclusion criteria were: pregnancy; severe underlying cardiopulmonary disease; coagulation disorders; concurrent non-neurological malignancies; participation in other interventional studies; discharge or death within 7 days post-tracheostomy; voluntary withdrawal. Based on an estimated decannulation success rate of 60% (10–14) and the Events Per Variable (EPV) method with EPV set at 10, the required sample size for the training set was ≥125 cases. According to a 7:3 ratio, the validation set required ≥54 cases.

### 2.2 Standardized decannulation protocol

Prerequisites for decannulation were: (1) off mechanical ventilation for ≥48 h; (2) hemodynamically stable (no vasoactive drugs); (3) SpO_2_ ≥95% on room air; (4) no signs of active infection; (5) informed consent obtained.

Capping trial: before decannulation, all patients underwent a 24-h full occlusion capping trial with close monitoring of respiration and oxygenation. The trial was completed immediately prior to the decannulation attempt, and its outcome was recorded before tube removal.

Decannulation method: before decannulation, emergency equipment was prepared at the bedside. After suctioning oral and airway secretions, the tracheostomy tube was quickly removed. Vital signs and oxygenation were closely monitored for 48 h post-decannulation.

Outcome definition: successful decannulation was defined as stable vital signs within 48 h after the first attempt, SpO_2_ consistently above 90%, no symptoms such as chest tightness or dyspnea, no worsening of the primary disease or new-onset pneumonia, and no need for reintubation or repeat tracheostomy (20, 21). Failed decannulation was defined as a composite outcome including early failure and late failure. Early failure was defined as reintubation or repeat tracheostomy within 48 h. Late failure was defined as discharge with the tracheostomy tube in place without meeting decannulation criteria, or remaining tracheostomized in-hospital for >90 days.

### 2.3 Data collection and organization

This study was approved by the Ethics Committee of the Affiliated Hospital of North Sichuan Medical College (Approval No.: 2023ER025-1). A data collection form was designed based on literature review and expert opinions. Data collection commenced from the day of tracheostomy. Data on decannulation indicators were based on records from within 24 h before the planned decannulation or on the day of discharge. Two individuals independently entered the data, and reliability and consistency checks were performed (Kappa ≥0.8). The proportion of missing data for each variable was < 5%. For variables with ≤ 10% missingness, mean imputation was used. Given the low missing rate, mean imputation was applied.

### 2.4 Statistical analysis

Statistical analysis was performed using SPSS 26.0 and R 4.3.0. Normally distributed continuous data were presented as mean ± standard deviation and compared using the t-test. Non-normally distributed data were presented as median (*M*) and interquartile range (P25, P75) and analyzed using rank-sum tests. Categorical data were presented as frequencies (*n*) and percentages (%) and compared using the χ^2^ test, corrected χ^2^ test, or Fisher's exact test. *P* < 0.05 was considered statistically significant.

Dichotomization of continuous predictors: Several continuous predictors were dichotomized for clinical interpretability. Lesion location was classified as supratentorial vs. infratentorial based on neuroanatomical specificity (infratentorial lesions involve the brainstem with higher risk of respiratory impairment). The cut-offs for GCS (>8) and albumin (≥35 g/L) followed established clinical thresholds. The Youden index yielded optimal values of 9.5 for GCS and 33.9 g/L for albumin, which closely approximated the clinically defined thresholds. Thresholds for airway suctioning frequency (< 6/24 h) and capping trial (≥24 h) were derived from previous studies (12, 20). This dichotomization facilitates bedside implementation while maintaining neurological specificity.

Variables with *P* < 0.10 in univariate analysis were included in multivariate logistic regression analysis using backward stepwise selection for exploratory variable identification. The Receiver Operating Characteristic (ROC) curve was plotted to assess model discrimination, and the area under the curve (AUC) was calculated. The Hosmer-Lemeshow test and calibration plots were used to evaluate model calibration. The Bootstrap method was used for internal validation. Decision Curve Analysis (DCA) was used to evaluate clinical utility. Based on the partial regression coefficients (β) of the indicators in the final logistic regression model, scores were calculated using the formula (score = β/smallest β × 2, rounded to the nearest integer) to establish the predictive scoring system. The optimal cut-off value was determined using the Youden index.

## 3 Results

### 3.1 General characteristics of study subjects

A total of 216 patients were included. The overall decannulation success rate was 63.9%. The average length of hospital stay was 50 days, with successful decannulation patients having an average cannulation duration of 32 days. The training set comprised 150 patients (success rate 66.0%), and the validation set comprised 66 patients (success rate 59.1%).

### 3.2 Univariate analysis

Univariate analysis showed that variables including Acute Physiology and Chronic Health Evaluation II (APACHE II), Charlson Comorbidity Index (CCI), Glasgow Coma Scale (GCS) at admission, lesion location, ICU length of stay, GCS at decannulation, number of airway suctioning episodes/24 h, capping trial, hemoglobin (Hb), and serum albumin (Alb) had *P* < 0.1 and were included in the multivariate logistic regression analysis (Table 1).

**Table 1:** Univariate analysis of factors associated with decannulation success in the training set (*N* = 150).

Variable	Successful decannulation (*n* = 99)	Failed decannulation (*n* = 51)	*χ^2^*	*P*
Gender			0.117	0.732
Male	63 (63.64)	31 (60.78)		
Female	36 (36.36)	20 (39.22)		
Age (years)			1.969	0.374
< 60	41 (65.08)	22 (34.92)		
60–74	46 (70.77)	19 (29.23)		
≥75	12 (54.55)	10 (45.45)		
BMI			3.299	0.192
< 18.5	4 (4.04)	5 (9.80)		
18.5–23.9	69 (69.70)	29 (56.86)		
≥24.0	26 (26.26)	17 (33.33)		
History of hypertension			0.365	0.546
Yes	67 (67.68)	32 (62.75)		
No	32 (32.32)	19 (37.25)		
History of stroke			1.244	0.265
Yes	11 (11.11)	9 (17.65)		
No	88 (88.89)	42 (82.35)		
History of chronic lung disease			0.672^*^	0.412
Yes	9 (9.09)	2 (3.92)		
No	90 (90.91)	49 (96.08)		
Smoking history			0.001	0.975
Yes	25 (25.25)	13 (25.49)		
No	74 (74.75)	38 (74.51)		
APACHEII score			5.070	0.024
< 15	46 (46.46)	14 (27.45)		
≥15	53 (53.54)	37 (72.55)		
CCI			2.784	0.095
< 6	55 (55.56)	21 (41.18)		
≥6	44 (44.44)	30 (58.82)		
GCS at admission			3.162	0.075
≤ 8	53 (53.54)	35 (68.63)		
>8	46 (46.46)	16 (31.37)		
Type of brain injury			0.277	0.871
Cerebrovascular disease	72 (72.73)	35 (68.63)		
Traumatic brain injury	22 (22.22)	13 (25.49)		
Brain tumor	5 (5.05)	3 (5.88)		
Lesion location			4.676	0.031
Supratentorial	86 (86.87)	37 (72.55)		
Infratentorial/combined	13 (13.13)	14 (27.45)		
Craniotomy			0.085	0.771
Yes	74 (74.75)	37 (72.55)		
No	25 (25.25)	14 (27.45)		
Decompressive craniectomy			0.000	0.994
Yes	31 (31.31)	16 (31.37)		
No	68 (68.69)	35 (68.63)		
Duration of endotracheal intubation (days)			2.474	0.116
≤ 5	81 (81.82)	36 (70.56)		
>5	18 (18.18)	15 (29.41)		
Tracheostomy timing			1.246	0.264
Early tracheostomy	58 (58.59)	25 (49.02)		
Late tracheostomy	41 (41.41)	26 (50.98)		
Duration of mechanical ventilation (days)			0.973	0.333
≤ 5	68 (68.69)	31 (60.78)		
>5	31 (31.31)	20 (39.22)		
ICU length of stay (days)			9.951	0.002
≤ 14	69 (69.70)	22 (43.14)		
>14	30 (30.30)	29 (56.86)		
Hyperbaric oxygen therapy (days)			0.063	0.802
≥10	37 (37.37)	18 (35.29)		
< 10	62 (62.63)	33 (64.71)		
GCS at decannulation			57.323	< 0.001
≤ 8分	5 (5.05)	31 (60.78)		
>8分	94 (94.95)	20 (39.22)		
Airway suctioning episodes/24 h			60.296	< 0.001
< 6 times/24 h	94 (94.95)	19 (37.25)		
≥6 times/24 h	5 (5.05)	32 (62.75)		
FOIS grade			0.876^*^	0.349
1–3	92 (92.93)	50 (98.04)		
4–7	7 (7.07)	1 (1.96)		
Capping trial			35.378	< 0.001
≥24 h	60 (60.61)	5 (9.80)		
< 24 h	39 (39.39)	46 (90.20)		
Hb (g/L)			9.166	0.010
≤ 89	13 (13.13)	17 (33.33)		
90–119	65 (65.66)	28 (54.90)		
≥120	21 (21.21)	6 (11.75)		
Alb (g/L)			9.155	0.002
< 35	31 (31.31)	29 (56.86)		
≥35	68 (68.69)	22 (43.14)		

^*^Corrected χ^2^ test used. Bold values: *P* < 0.1 (variables selected for multivariate analysis).

### 3.3 Establishment of the logistic regression model

Collinearity analysis showed no significant multicollinearity among the variables (22). Multivariate logistic regression analysis revealed that supratentorial lesions, GCS >8 at decannulation, < 6 airway suctioning episodes/24 h, capping trial ≥24 h, and Alb ≥35 g/L were independent predictive factors for successful decannulation (Table 2).

**Table 2:** Multivariate logistic regression analysis of predictors for successful decannulation.

Variable	β	SE	Wald	*P*	*OR*	95%CI
Lesion location						
Supratentorial	2.085	0.746	7.814	0.005	8.047	1.865–34.720
Infratentorial/combined	—	—	—	—	—	—
GCS at decannulation						
≤ 8	—	—	—	—	—	—
>8	3.054	0.749	16.636	< 0.001	21.209	4.887–92.036
Airway suctioning/24 h						
< 6 times/24 h	2.575	0.703	13.401	< 0.001	13.126	3.307–52.093
≥6 times/24 h	—	—	—	—	—	—
Capping trial						
< 24 h	—	—	—	—	—	—
≥24 h	1.838	0.693	7.038	0.008	6.283	1.616–24.426
Alb						
< 35 g/L	—	—	—	—	—	—
≥35 g/L	1.550	0.608	6.495	0.011	4.710	1.430–15.511
Constant	−6.636	1.369	23.481	< 0.001	0.001	—

β, partial regression coefficient; SE, standard error; Wald, Wald chi-square test; OR, odds ratio; CI, confidence interval.

**Figure 1:**
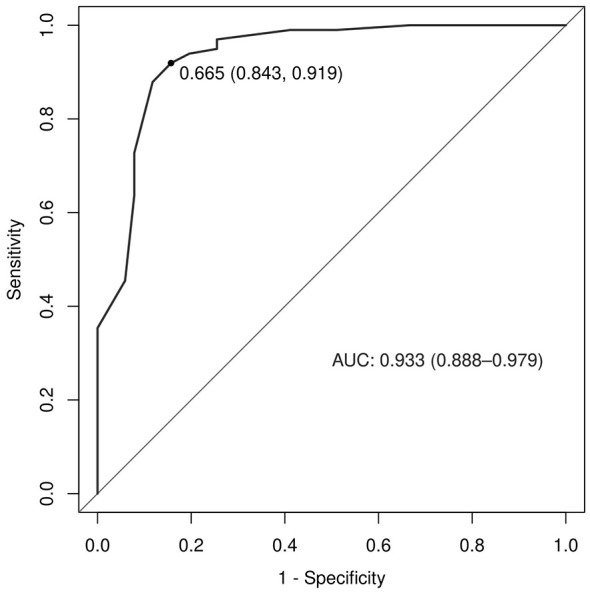
ROC curve of the predictive model.

**Figure 2:**
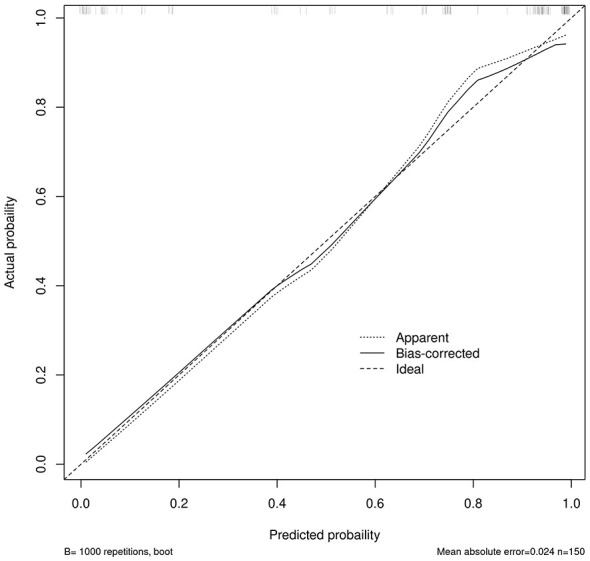
Calibration curve of the prediction model.

**Figure 3:**
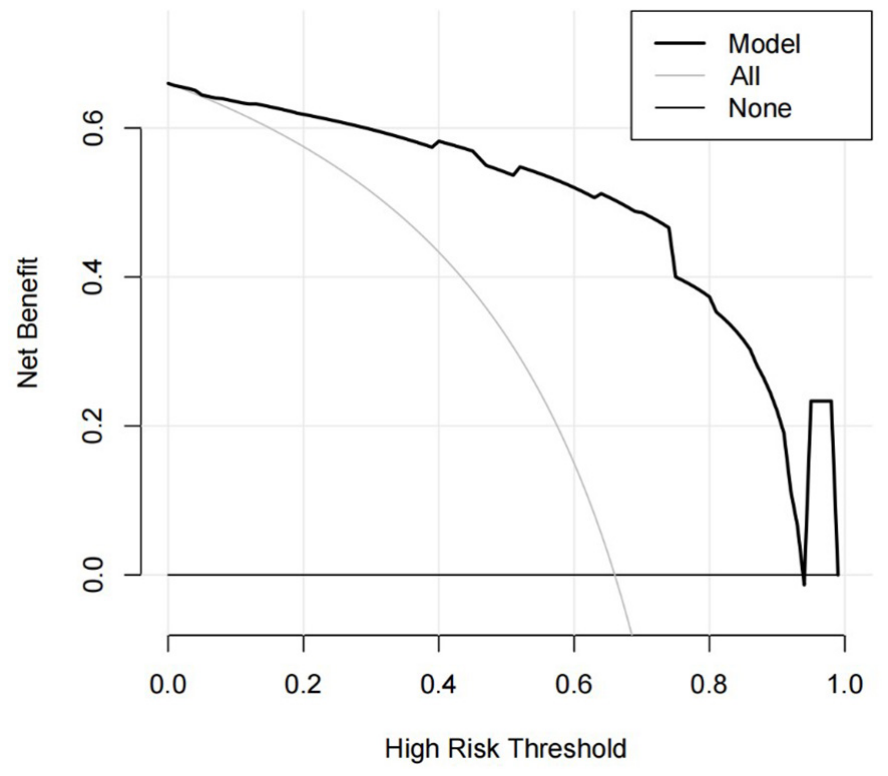
DCA curve of the prediction model.

Based on the β values, the logistic equation was constructed: Logit*P* = −6.636 + 2.085 × Lesion Location + 3.054 × GCS at Decannulation + 2.575 × Airway Suctioning/24 h + 1.838 × Capping Trial + 1.550 × Alb. The ROC curve for the model showed an AUC of 0.933 (Figure 1). The calibration plot showed the observed curve close to the ideal diagonal line (Figure 2). The calibration slope was 1.008, the intercept was −0.005, and the Brier score was 0.085, indicating good model calibration. The Hosmer-Lemeshow test further supported this (χ^2^ = 10.757, *P* = 0.096). DCA for the training set (Figure 3) showed that the model provided a positive net benefit across thresholds of 0–0.85, with the curve consistently above the “treat all” and “treat none” strategies, indicating higher net benefit when guided by the model. Net benefit decreased with fluctuation at thresholds >0.85, which is typical near extreme values.

Stepwise logistic regression was used for exploratory variable selection, which carries a risk of overfitting. To assess model stability, we performed internal validation using bootstrap resampling (1,000 repetitions). The optimism-corrected c-index was 0.922, close to the apparent c-index of 0.933, indicating minimal overfitting. No additional shrinkage methods were applied, as the bootstrap results suggested adequate stability.

### 3.4 Establishment and evaluation of the predictive scoring system

Based on the β values, scores were assigned as 3, 4, 3, 3, and 2 for the respective variables (Table 3). The total score ranged from 0 to 15 points. The optimal cut-off value determined by the Youden index was 9.5 points, rounded to 10 points. A score ≥10 indicated a high probability of successful decannulation, and a score < 10 indicated a low probability. The scoring system demonstrated a sensitivity of 91.9%, specificity of 84.3%, and accuracy of 89.3%, positive predictive value of 91.9% and negative predictive value of 84.3% in the training set (Table 4). Comparison of the AUCs of the logistic regression model and the scoring system showed no statistically significant difference (*Z* = 0.261, *P* = 0.794), suggesting that the scoring system could replace the regression model for predicting decannulation success.

**Table 3:** Scoring criteria based on the logistic model for successful decannulation.

Variable	β	Assigned score
Lesion location (supratentorial)	2.085	3
GCS at decannulation (>8)	3.054	4
Airway suctioning episodes (< 6/24 h)	2.575	3
Capping trial (≥24 h)	1.838	3
Alb (≥35 g/L)	1.550	2

**Table 4:** Prediction of decannulation success using the scoring system in the training set.

Predicted outcome	Successful decannulation (observed)	Failed decannulation (observed)	Total
Low success probability (score < 10)	8	43	51
High success probability (score ≥10)	91	8	99
Total	99	51	150

Sensitivity = true positives/(true positives + false negatives) ^*^ 100%;

Specificity = true negatives/(true negatives + false positives) ^*^ 100%;

Accuracy = (true positives + true negatives)/total ^*^ 100%.

### 3.5 Temporal validation

**Figure 4:**
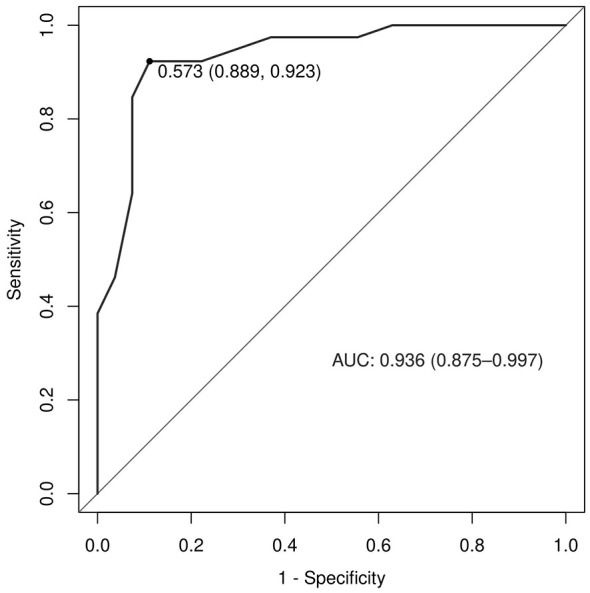
ROC curve of the prediction model on the validation set.

**Figure 5:**
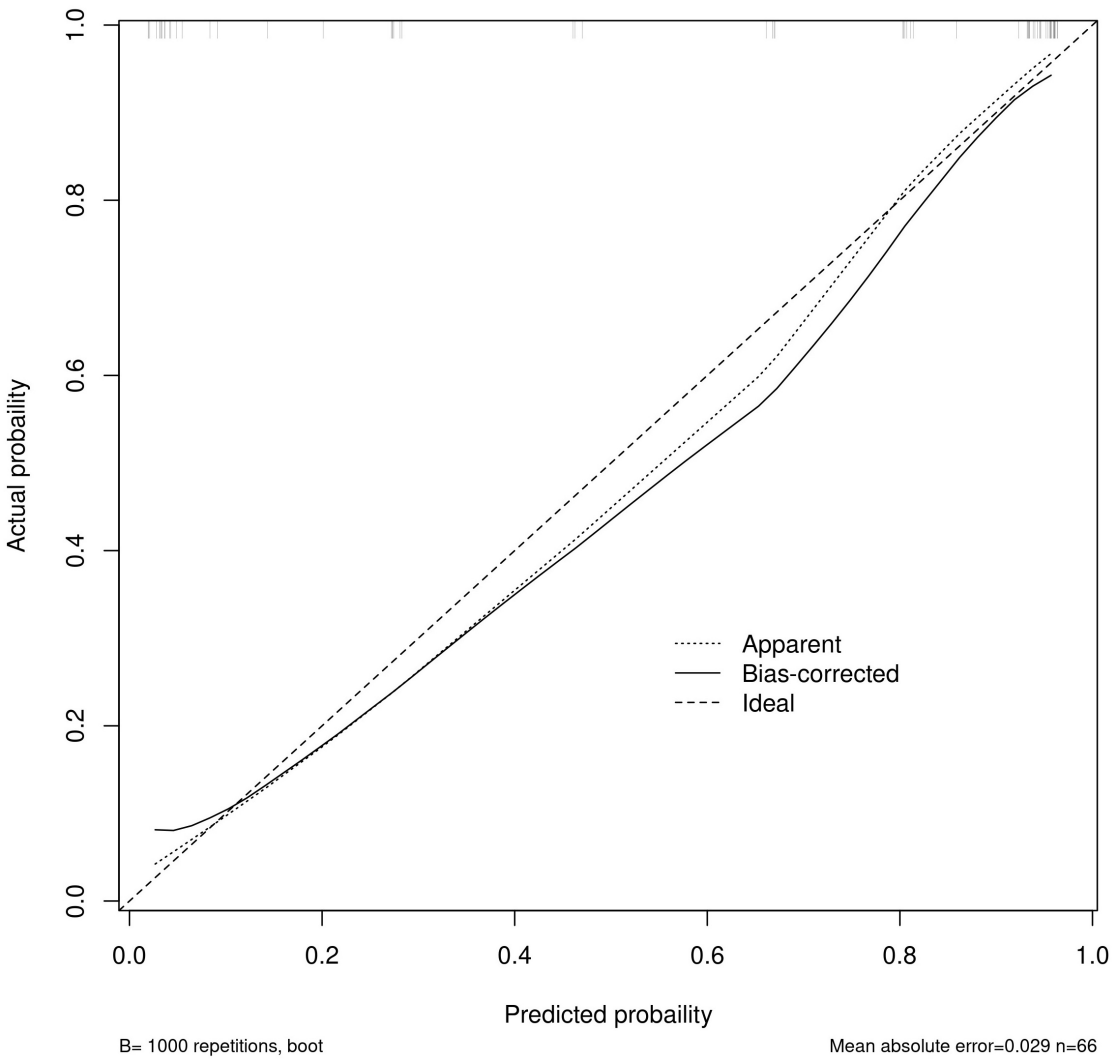
Calibration curve of the predictive model on the validation set.

**Figure 6:**
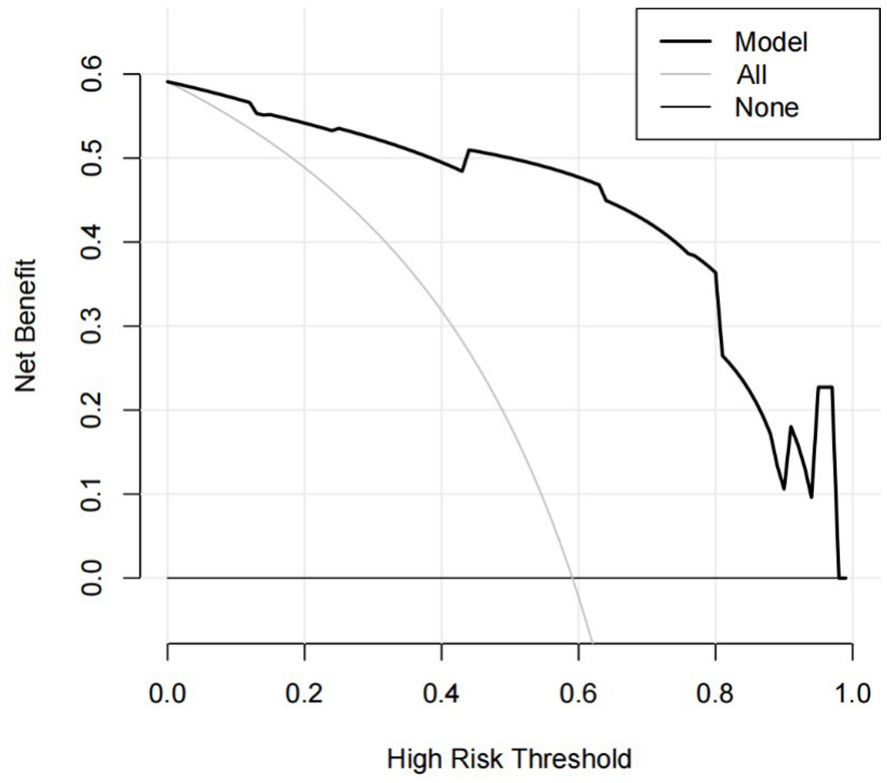
DCA curve of the prediction model on the validation set.

In the validation set, the logistic regression model showed an AUC of 0.936 (95% CI: 0.875–0.997; Figure 4). The calibration slope was 1.024, the intercept was −0.033, and the Brier score was 0.096, indicating good model calibration and predictive accuracy, and the Hosmer-Lemeshow test yielded χ^2^ = 7.778, *P* = 0.169 (Figure 5). The validation set DCA (Figure 6) mirrored the training set, showing positive net benefit across 0–0.85 and supporting generalizability; minor fluctuations at >0.9 reflect the smaller sample size. Using a cut-off of 10 points, the scoring system achieved an AUC of 0.930 (95% CI: 0.868–0.993), with a sensitivity of 84.6%, specificity of 92.6%, and accuracy of 87.9%, positive predictive value of 94.3% and negative predictive value of 80.6% (Table 5). Comparison of the AUCs between training and validation sets showed no statistically significant difference (*Z* = 0.358, *P* = 0.720), indicating good generalizability.

**Table 5:** Prediction of decannulation success using the scoring system in the validation set.

Predicted outcome	Successful decannulation (observed)	Failed decannulation (observed)	Total
Low success probability (score < 10)	6	25	31
High success probability (score ≥10)	33	2	35
Total	39	27	66

## 4 Discussion

### 4.1 Decannulation success rate

This study found an overall decannulation success rate of 63.9%, consistent with previous research (11, 12). Reported success rates vary in the literature: a 2019 meta-analysis reported rates ranging from 46.7% to 97.5% in neurocritical patients (12), and a 2021 systematic review by Wahlster et al. reported rates of 54.7% to 73.6% in brain-injured patients (11).

### 4.2 Independent predictive factors

The identified independent predictive factors—supratentorial lesion location, GCS >8 at decannulation, < 6 airway suctioning episodes/24 h, capping trial ≥24 h, and Alb ≥35 g/L—are consistent with previous studies (20, 23–25). Supratentorial lesions are associated with better prognosis compared to infratentorial lesions, which affect respiratory centers and the swallowing central pattern generator (26–29). GCS is an established predictor of decannulation success (30–32), with a threshold of >8 widely used in clinical practice. Airway secretion burden, reflected by suctioning frequency, is a key indicator of cough effectiveness and airway clearance (21, 33). The capping trial remains a standard final step in decannulation assessment (20, 25, 34). Alb reflects nutritional status, which is critical for recovery and successful decannulation (2, 35–38).

### 4.3 Clinical utility and comparison with existing tools

Compared with traditional clinical judgment, which relies heavily on individual experience and subjective assessment, our scoring system provides a standardized, quantitative, and reproducible tool for decannulation decision-making. It reduces inter-clinician variability and can be easily applied at the bedside.

Compared with existing prediction tools, the DECAN score developed by Park et al. (7) achieved an AUC of 0.890, with a specificity of 84.6% and sensitivity of 80.2%. The QsQ score established by Santus et al. (25) included both quantitative and semi-quantitative indicators but lacked a clear basis for score assignment and was not limited to the neurosurgical population. The study by Enrichi et al. (20) focused primarily on airway-related factors. Schneider et al. (23) developed a multivariate model incorporating variables such as age and lesion location. In contrast, the present study was a prospective observational study that included 216 neurosurgical patients. Its indicators are highly specific to the target population, and the scoring system demonstrated good discrimination and accuracy. The scoring system also includes lesion location (supratentorial vs. infratentorial), a neurological factor that is particularly relevant to neurosurgical patients but often overlooked in general ICU prediction tools.

### 4.4 Clinical implications of false-positive and false-negative predictions

False-positive results (score ≥10 but decannulation fails) may cause premature decannulation, leading to reintubation, hypoxia, aspiration, and prolonged hospital stay. False-negative results (score < 10 but decannulation would succeed) may lead to unnecessary tube retention, delayed recovery, increased infection risk, and higher nursing burden. Thus, the scoring system should support, not replace, clinical judgment. Combining it with bedside assessments (e.g., cough strength, swallowing function) can reduce misclassification risks. Future studies should explore optimal thresholds for different patient subgroups.

### 4.5 Limitations

This study has several limitations. First, the sample size was relatively modest and originated from only two institutions, which may affect generalizability. Moreover, the validation cohort was derived from the same two institutions as the training cohort, separated only by time; therefore, this is a temporal validation rather than a true external validation. This approach may overestimate model performance due to shared population characteristics. Second, the study scope was limited to hospitalized patients, lacking data from home care and community settings. Third, influenced by the regional economic level, some patients who withdrew from treatment early were not included, which may have introduced bias. Fourth, only five predictors were included, and other potentially important factors such as quantitative cough strength or detailed swallowing assessment were not evaluated. Fifth, mean imputation was used for missing data rather than more robust approaches such as multiple imputation. Although the overall missing rate was low (< 5%), this method may not fully account for the uncertainty associated with missing values, potentially leading to biased estimates or reduced statistical power. Sixth, dichotomizing continuous predictors (e.g., GCS, albumin) may reduce statistical power, but the model still showed good discrimination. Seventh, the composite outcome combined early and late failures, which may have distinct predictors. However, the limited number of events precluded separate analyses; future studies should address this. Eighth, the capping trial may introduce incorporation bias because it is part of the decannulation decision. This reflects real-world clinical practice, but future studies without this predictor could help assess this bias. Future multicenter studies with larger samples, additional predictors, and more robust missing data methods are needed to further validate and improve the tool.

## 5 Conclusion

Through this prospective observational clinical study, we identified supratentorial lesion location, GCS >8 at decannulation, < 6 airway suctioning episodes/24 h, capping trial ≥24 h, and Alb ≥35 g/L as independent predictive factors for successful tracheostomy decannulation in neurosurgical patients. The prediction model and scoring system constructed based on these factors demonstrate good predictive performance. This scoring system provides a simple, practical bedside tool to help clinicians identify patients suitable for decannulation, potentially improving success rates and reducing complications.

## Data Availability

The raw data supporting the conclusions of this article will be made available by the authors, without undue reservation.

## References

[1] The Writing Committee of the Report on Cardiovascular Health and Diseases in China. Report on cardiovascular health and diseases in China 2022: an updated summary. Biomed Environ Sci. (2023) 36:669–701. doi: 10.3967/bes2023.106 37711081

[2] Neurosurgery Neurosurgery Branch of Chinese Medical Association Chinese Neurosurgery Critical Care Management Collaboration Group. Expert consensus on airway management for critically ill neurosurgical patients in China (2016). Natl Med J China. (2016) 96:1639–42. doi: 10.3760/cma.j.issn.0376-2491.2016.021.004

[3] Bosel J. Use and timing of tracheostomy after severe stroke. Stroke. (2017) 48:2638–43. doi: 10.1161/STROKEAHA.117.017794 28733479

[4] Krishnamoorthy V Hough CL Vavilala MS Komisarow J Chaikittisilpa N Lele AV . Tracheostomy after severe acute brain injury: trends and variability in the USA. Neurocrit Care. (2019) 30:546–54. doi: 10.1007/s12028-019-00697-5 30919303 PMC6582655

[5] Lei L Wu CW Chen W Luo XH. Analysis of predictive factors for tracheostomy in neurocritical patients. J Clin Emerg. (2022) 23:147–50. doi: 10.13201/j.issn.1009-5918.2022.02.014

[6] Chen Q Wang JD. Clinical progress in percutaneous tracheostomy. Chin J Otorhinolaryngol Head Neck Surg. (2010) 45:342−5. doi: 10.3760/cma.j.issn.1673-0860.2010.04.023 20627063

[7] Park C Ko RE Jung J Na SJ Jeon K. Prediction of successful de-cannulation of tracheostomised patients in medical intensive care units. Respir Res. (2021) 22:131. doi: 10.1186/s12931-021-01732-w 33910566 PMC8080087

[8] Coplin WM Pierson DJ Cooley KD Newell DW Rubenfeld GD. Implications of extubation delay in brain-injured patients meeting standard weaning criteria. Am J Respir Crit Care Med. (2000) 161:1530–6. doi: 10.1164/ajrccm.161.5.9905102 10806150

[9] Quiñones-Ossa GA Durango-Espinosa YA Padilla-Zambrano H Ruiz J Moscote-Salazar LR Galwankar S . Current status of indications, timing, management, complications, and outcomes of tracheostomy in traumatic brain injury patients. J Neurosci Rural Pract. (2020) 11:222–9. doi: 10.1055/s-0040-1709971 32367975 PMC7195963

[10] Heidler M Salzwedel A Jöbges M Lück O Dohle C Seifert M . Decannulation of tracheotomized patients after long-term mechanical ventilation - results of a prospective multicentric study in German neurological early rehabilitation hospitals. BMC Anesthesiol. (2018) 18:65. doi: 10.1186/s12871-018-0527-3 29898662 PMC6000940

[11] Wahlster S Sharma M Chu F Granstein JH Johnson NJ Longstreth WT . Outcomes after tracheostomy in patients with severe acute brain injury: a systematic review and meta-analysis. Neurocrit Care. (2021) 34:956–67. doi: 10.1007/s12028-020-01109-9 33033959 PMC8363498

[12] Ding YJ Xu SX Zhang W Zhang B A. meta-analysis of clinical indications for extubation in neurocritical patients with tracheostomy. Chin J Crit Care Med. (2019) 31:1378–83. doi: 10.3760/cma.j.issn.2095-4352.2019.11.013 31898569

[13] Song L Wang Y Li HD Liu J Li Z Zhang WJ . Factors associated with successful decannulation in patients with severe acquired brain injury. Chin J Stroke. (2021) 16:699–704. doi: 10.3969/j.issn.1673-5765.2021.06.011

[14] He YB Zhou XJ Lin QM Cao CY Xie QY. Analysis of factors affecting decannulation in patients with chronic disturbance of consciousness complicated by tracheostomy. J Third Mil Med Univ. (2021) 43:1444–8. doi: 10.16016/j.1000-5404.202102109

[15] Ba CH Ping P Zhang HX Liu C Wang YW. Pulmonary infection and quality of life in long-term tracheostomy patients. J Prev Med Chin PLA. (2017) 35:498–500, 503. doi: 10.13704/j.cnki.jyyx.2017.05.024

[16] Cordeiro A Santos J Barroso A Donoso M Mata L Chianca T. Tracheostomy care for adults and the elderly in the home environment: a scoping review. Rev Esc Enferm USP. (2024) 58:e20240028. doi: 10.1590/1980-220X-REEUSP-2024-0028en 39101811 PMC11299533

[17] Yin SH Peng Y Zhong LL Shi ZY. Meta-integration of qualitative research on caregiving experiences of caregivers of tracheostomy patients. Chin J Mod Nurs. (2021) 27:3251–8. doi: 10.3760/cma.j.cn115682-20210113-00185

[18] Zhou Z Wang W Li Y Jin K Wang X Wang Z . In-depth mining of clinical data: the construction of clinical prediction model with R. Ann Transl Med. (2019) 7:796. doi: 10.21037/atm.2019.08.63 32042812 PMC6989986

[19] Collins GS Reitsma JB Altman DG Moons KGM. Transparent reporting of a multivariable prediction model for individual prognosis or diagnosis (TRIPOD): the TRIPOD statement. BMJ. (2015) 350:g7594. doi: 10.1136/bmj.g7594 25569120

[20] Enrichi C Battel I Zanetti C Koch I Ventura L Palmer K . Clinical criteria for tracheostomy decannulation in subjects with acquired brain injury. Respir Care. (2017) 62:1255–63. doi: 10.4187/respcare.05470 28698267

[21] Stelfox HT Crimi C Berra L Noto A Schmidt U Bigatello LM . Determinants of tracheostomy decannulation: an international survey. Crit Care. (2008) 12:R26. doi: 10.1186/cc6802 18302759 PMC2374629

[22] O'brien RM. A caution regarding rules of thumb for variance inflation factors. Qual Quant. (2007) 41:673–90. doi: 10.1007/s11135-006-9018-6

[23] Schneider H Hertel F Kuhn M Ragaller M Gottschlich B Trabitzsch A . Decannulation and functional outcome after tracheostomy in patients with severe stroke (DECAST): a prospective observational study. Neurocrit Care. (2017) 27:26–34. doi: 10.1007/s12028-017-0390-y 28324263

[24] Hernández Martínez G Rodriguez ML Vaquero MC Ortiz R Masclans JR Roca O . High-flow oxygen with capping or suctioning for tracheostomy decannulation. N Engl J Med. (2020) 383:1009–17. doi: 10.1056/NEJMoa2010834 32905673

[25] Santus P Gramegna A Radovanovic D Raccanelli R Valenti V Rabbiosi D . A systematic review on tracheostomy decannulation: a proposal of a quantitative semiquantitative clinical score. BMC Pulm Med. (2014) 14:201. doi: 10.1186/1471-2466-14-201 25510483 PMC4277832

[26] Qureshi AI Suarez JI Parekh PD Bhardwaj A. Prediction and timing of tracheostomy in patients with infratentorial lesions requiring mechanical ventilatory support. Crit Care Med. (2000) 28:1383–7. doi: 10.1097/00003246-200005000-00020 10834682

[27] Wu YX Wang Q. Introduction to scoring system for extubation in postoperative neurosurgical patients. Chin J Emerg Resusc Disaster Med. (2011) 6:665–6. doi: 10.3969/j.issn.1673-6966.2011.07.033

[28] Qiao J Wu ZM Ye QP Dai M Dai Y He ZT . Characteristics of dysphagia among different lesion sites of stroke: a retrospective study. Front Neurosci. (2022) 16:944688. doi: 10.3389/fnins.2022.944688 36090270 PMC9449127

[29] Kim YK Cha JH Lee KY. Comparison of dysphagia between infratentorial and supratentorial stroke patients. Ann Rehabil Med. (2019) 43:149–55. doi: 10.5535/arm.2019.43.2.149 31072081 PMC6509573

[30] Medeiros GCD Sassi FC Lirani-Silva C Andrade CRFD. Critérios para decanulação da traqueostomia: revisão de literatura. CoDAS. (2019) 31:e20180228. doi: 10.1590/2317-1782/20192018228 31800881

[31] Li SM Wu XY Wang J Zeng JR Lei ZJ Du CP . Research progress on decannulation management in tracheostomized patients. West China Med J. (2022) 37:783–7.

[32] Mehta R Chinthapalli K. Glasgow coma scale explained. BMJ. (2019) 365:l1296. doi: 10.1136/bmj.l1296 31048343

[33] Ingrams D Mcgrath B Narula T . Tracheostomy Guidelines for NHS Wales Adults and Children [Internet]. (2019). Available online from: https://fdocuments.net/document/tracheostomy-guidelines-for-nhs-wales.html?page=1 (Accessed October 1, 2023).

[34] Korean Bronchoesophagological Society Guideline Task Force Nam IC Shin YS Jeong WJ Park MW Park SY Song CM . Guidelines for tracheostomy from the Korean bronchoesophagological society. Clin Exp Otorhinolaryngol. (2020) 13:361–75. doi: 10.21053/ceo.2020.00353 32717774 PMC7669309

[35] Ni J X Fang CG Li Y. Analysis of clinical characteristics of failed extubation in critically ill tracheostomized patients. Chin J Integr Tradit West Med Intensive Crit Care. (2017) 24:608–12. doi: 10.3969/j.issn.1008-9691.2017.06.011

[36] Lu CJ An HW Wei BX. Study on factors influencing extubation in critically ill neurological patients with tracheostomy. J Clin Exp Med. (2014) 13:976–9. doi: 10.3969/j.issn.1671-4695.2014.12.009

[37] Wang SW Zeng X Li QF Wang LG Li H P. Factors associated with decannulation in patients with persistent vegetative state complicated by tracheostomy. Chin J Phys Med Rehabil. (2022) 44:907–11. doi: 10.3760/cma.j.issn.0254-1424.2022.10.009

[38] Neurosurgery Neurosurgery Branch of Chinese Medical Association Chinese Neurosurgery Critical Care Management Collaboration Group. Expert consensus on nutritional therapy for critically ill neurosurgical patients in China (2022 edition). Natl Med J China. (2022) 102:2236–55. doi: 10.3760/cma.j.cn112137-20220621-01362

